# Developmental and Cognitive Characteristics of “High-Level Potentialities” (Highly Gifted) Children

**DOI:** 10.1155/2011/420297

**Published:** 2011-10-01

**Authors:** Laurence Vaivre-Douret

**Affiliations:** ^1^University of Paris Descartes, Sorbonne Paris Cité, and Inserm UMR-S0669 University Paris-Sud-Paris Descartes, 12 Rue de l'École de Médecine, 75006 Paris, France; ^2^Department of Obstetric and Gynecology, AP-HP Port Royal-Cochin Hospital, 123 Boulevard de Port-Royal, 75014 Paris, France; ^3^Necker-Enfants Malades Hospital, Inserm Unit 669, 149 Rue de Sèvres, 78743 Paris Cedex 15, France

## Abstract

This study covers the interesting field of the development in gifted children which is often neglected in pediatrics because psychomotor development data are still rare, since “gifted” children are generally noticed towards the end of their primary schooling by IQ measurement. Developmental studies have shown the evidence from several fields that children identified as “high-level potentialities” or “intellectually gifted” develop sensory, locomotor, neuropsychological, and language skills earlier than typically expected. The hypothesis is offered that the earlier development originates from biological processes affecting the physical development of the brain and in turn even intellectual abilities are developed earlier, potentially allowing for advanced development. Further it is discussed how these developmental advances interact with the social environment and in certain circumstances may entail increased risk for developing socioemotional difficulties and learning disabilities that often go unaddressed due to the masking by the advance intellectual abilities.

## 1. Introduction

Since the 19th century, children showing particular potential, often called “gifted”, have aroused interest among authors such as Lombrosso [1] in the area of the links between genius and madness, or in differential psychology [2]. 

 As early as 1909, Alfred Binet, the originator of the notion of “mental age,” noted schoolchildren who were “too intelligent” and who derived no benefit from education.

 Over the 20th century, different psychiatrists and psychologists took a particular interest in these exceptionally gifted children, in particular Terman in 1925 [3] who conducted a longitudinal study over 35 years starting in 1922. He studied 1528 subjects (671 girls and 857 boys) whose mean IQ was 150 (range 135 to 200) and mean age 11 years at the start of the survey. He was able to identify characteristics that were common to the sample overall. The aim was to obtain better information about this particular population so as to implement educational measures. Since this work, the identification of gifted children has led to various lines of research into superior intelligence. However little work has been conducted on relationships with neuropsychomotor development, while more numerous are those studies that have focused on understanding the cognitive functioning of these children at school age. More recently, research in developmental neuropsychology has shown the cerebral activation occurring in the course of intellectual tasks, which has opened up interesting possibilities.

 Particularly, able children are variously referred to in the literature as “gifted,” “highly gifted,” “precocious,” “early developers,” “intellectually precocious,” “talented," “bright," “geniuses,” “prodigies,” and so forth, or as having “high-level potentialities” or “high intellectual potential”.

 These various terms are not necessarily synonymous, since they possess differing connotations and relate to differing theoretical conceptions. “Precocious,” for instance, adds the notion of developmental dating in early childhood, restricts the notion to children, and excludes any consideration of adults. Phrases such as “intellectually precocious” or “high intellectual potential” focus solely on the intellectual domain setting aside other functions, whether bodily, praxis, or affective. The French term “*surdoué*” synonymous of exceptionally or “highly gifted” in English suggests an individual who can cope with anything (like Superman) and who is not allowed to make mistakes, or be unsuccessful, and certainly not expected to experience learning difficulties. The term “*surdoué*” in French or “highly gifted”, like the notion of the “gifted” child with its connotation of having received the “gift” of intelligence, can lead to rather dangerous or passionate polemics, so that we have chosen to refer to these children as having “high-level potentialities”, or in fact as being “particularly able”. Indeed, if the child naturally possesses exceptional abilities, these are not connected with learning or education, and they set the child aside, qualitatively and quantitatively, from his or her peers. Thus the child's “potentialities” refer to potential skills, talents, and abilities that can be triggered to develop in certain circumstances that relate to issues of feasibility, implementation, and motivation [4, 5].

## 2. Identification of “High-Level Potentialities” (Highly Gifted) Children

 While various techniques are used to detect indicators of intellectual precocity (parental observation of the development and behaviour of the child, academic performance, teachers' observations, etc.), the only tool that is widely agreed upon to define this group of children is the Intelligence Quotient (IQ) which is determined from batteries of tests such as the WPPSI (Wechsler Preschool and Primary School Intelligence) scale [6], or the WISC (Wechsler Intelligence Scale for Children, [7]). No other objective criterion is used widely enough to serve as a reference. Thus a child is considered to belong to a “gifted” population if his or her IQ has proved to be exceptionally high on the basis of a rigorous evaluation conducted by a clinical psychologist.

 The first intelligence tests were developed in 1905 by two French psychologists, Binet and Simon, with the creation of the first “metric scale” for intelligence. This scale was adapted in 1916 by Terman, a Californian psychologist who as early as 1922 instated the largest-ever longitudinal study on gifted children. In 1911, Stern [10], a German psychologist, introduced the concept of IQ which is the ratio of a mental age to chronological age multiplied by 100. However, for children, the problem was the mental age, and it was thus that in 1930 Wechsler, an American psychologist, resorted to a method involving statistical standardisation that enabled the classification of the results of an individual not in terms of mental age but according to a given ranking in relation to results derived from the general population of the same age. He thus created the standard IQ measure (mean = 100, SD = 15) for different tests (WPPSI, WISC, WAIS (Wechsler Adult Intelligence Scale) [6, 7, 11].

 These scales [6, 7, 11] have recently been updated in France with the WPPSI-III (2 years 6 months to 7 years 4 months) in 2004, the WISC-IV (6 years to 16 years 11 months) in 2005, and the WAIS-III (16 years to 99 years) in 2000. The classification according to IQ in France is the only scale that is accepted by protagonists overall (medical, paramedical, psychologists, and teachers). However, opinions differ on the threshold beyond which the terms of “precocity” or “giftedness” should be applied. Levels beyond which these terms should be used range according to viewpoints from a score of 120 to 140 or even more. It is for instance fixed at 135 by Terman (International Encyclopedia of Education, p.2492) and at 120 in certain American states (see Encyclopedia Britannica), or again at 125 by the psychologist Terrassier [12, 13] founder of the French National foundation ANPEIP for gifted children. 

 According to the French Psychiatrist Ajuriaguerra [14], a “gifted” child is one who possesses superior abilities, well above those of other children of the same age; a “gifted” child obtains an IQ score of 140+ (quantitative measure) and presents exceptional personality traits from a qualitative point of view (creative talent in several domains). It is obvious that depending on the threshold used, the reference population is not the same.

 According to a recent report by Delaubier [15], commissioned by the French Ministry of Education, if a threshold of 120 is used, a large number of schoolchildren are taken into consideration (one or two per class). Beyond 145 (0.13% of the population and around one child in a thousand), the population considered is one of extremely exceptional individuals, too rare to constitute a “group” affording scope for statistical description or for the establishment of separate education facilities. The threshold of 130 (amounting to around 2.28% of the population or one child in forty) is the most commonly selected to consider that a child is “gifted”. On this basis, and again according to the Delaubier report [15], the numbers involved can be estimated in France at about 200 000 school children between the ages of 6 and 16, corresponding to the period of compulsory schooling.

 However, the measurement of intelligence is not sufficient on its own to predict later social or intellectual success, or the production of creative work [16]. There are interactions among high ability, task commitment, and creativity [17].

 Sternberg [18, 19] proposed a triangular theory of intelligence for “gifted” children (the “triarchic mind”): this involved analytical intelligence (measured by IQ), practical intelligence (deriving implicit rules from a given situation), and creative intelligence (producing original creations).

 In this perspective, the restriction of the term “gifted” to children obtaining a given IQ score appears as a highly arbitrary oversimplification.

 As a complement to the Wechsler test batteries, more focused “g-factor” tests [20, 21] have been used to remove the cultural register, such as Raven's Progressive Matrices or Antsey's domino test.

 Certain definitions of intellectual precocity, for instance, that given in the Marland report [22] in the USA, are designed to evidence particular aptitudes that may exist in isolation or in association. A multifaceted view of giftedness proposed by Marland [22] has been adopted by the US Department of Education and a majority of state departments of education and school systems. This report described gifted and talented children as those who demonstrate high achievement or potential in any one of six areas: specific academic aptitude, general intellectual ability, leadership ability, creative or productive thinking, visual and performing arts, and psychomotor ability (which was deleted in subsequent legislation). According to this report, gifted and talented children are recognised by professionally qualified individuals on the basis of exceptional ability and their potential for out-of-the-ordinary performances. A revised definition has asserted that “outstanding talents are present in children and youth from all cultural groups, across all economic strata and all areas of human endeavor” (US Department of Education, 1993, p.26).

 While the classic tests such as the Wechsler tests (e.g., WISC III) only measure three types of intelligence, linguistic, spatial, and logicomathematical, Gardner [23] in his “multiple intelligences” theory describes seven types of intelligence that are independent one from another: linguistic, logico-mathematical, visuospatial, musical, somatokinetic, interpersonal, and introspective. However, this modular approach is derived from studies on patients having sustained brain damage. In addition, Gardner also developed the nonquantifiable notion of the emotional quotient (EQ). EQ measures integrate the emotional and affective experience of the individual by way of specific tests.

 Although the methods used by Gardner [23] are open to criticism, this work is relatively recent and is in favour of not using IQ alone but of setting it alongside other tests approaching the psychoaffective sphere. This leads on to systematic complementary investigations including personality tests, and, depending on difficulties experienced on certain subtests, more advanced neuropsychological tests [24]. Deficits in certain functions can indeed lead the individual to overinvest in the intact functions by way of overcompensation, or indeed as a result of overstimulation.

 A further dimension emerges from the scientific literature devoted to precocity and talent, and that is creativity [25], with a few existing tests [26, 27]. Here, creativity can be defined as the ability to generate original productions that are suited to the requirements of the situation, the task, or the problem [28].

### 2.1. Intraindividual Variability and Complementary Evaluations

Besides the intelligence and performance tests that are in wide use to identify children with “high-level potentialities,” interviews and questionnaires are also frequently used, but they have not been subjected to any empirical validation procedure in France [13], whereas, in the USA, there are standardised scales and, in particular, the SRBCS (Scale for Rating the Behavioural Characteristics of Superior Students) [29].

 It has frequently been observed in intraindividual research on children with “high-level potentialities” using the Wechsler scales that differences occur between VIQ (verbal) and PIQ (performance) (12 points) although this difference is not always found to be significant [30]. However, intraindividual variability has been observed across various studies where VIQ may be significantly higher than PIQ [31] or on the contrary lower [32]. Vaivre-Douret [4, 5] wonders about the significance of VIQ/PIQ heterogeneity, which in her view should not be seen as being a specific characteristic of these children. Indeed, the significant difference in favour of the VIQ is enhanced by high performances in these children's verbal skills (frequently reaching saturation) in cases, where at the same time, they experience greater difficulty with performance tests. Indeed, in a child of average abilities, the difference is less marked between VIQ and PIQ in case of difficulty on the performance scale, because here VIQ scores do not reach as high a level as for children with “high-level potentialities.” Hence, there are differences that can be attributed to methodological bias between studies that recruit their “high-level potentialities” child populations in psychologist consultant populations [31, 33] rather than in the general population. In the latter case, there is a lesser risk in quantitative terms of finding children experiencing difficulties and therefore of finding large differences between VIQ and PIQ.

 Only complementary neuropsychological evaluations, as yet still rarely implemented, can give insight into this difference observed on certain subtests. Across the literature, it does appear that the diagnostic criteria used for identification purposes are not the subject of any consensus, and the IQ threshold score is also variable. In addition, apart from the IQ measure, there is wide variability in the complementary measures that are implemented. It is also important to take the “FLYNN effect” into account [34, 35] which concerns gifted children as well as the others. The FLYNN effect is a regular yearly increase of one third of a point in IQ between the construction of the standard and the examination date.

 If according to the literature different factors are involved in the emergence of exceptional ability or talent, it has also been shown that performance is sensitive to characteristics of the socioeconomic environment [36–39], in particular the environmental context provided by the parents [40, 41]. These children are less often detected in underprivileged environments [4]. According to different studies, other factors relate to cognitive and conative variables and to creativity factors [42–48].

## 3. Sensory-Motor and Cognitive Development

 From birth, biological maturing processes are continuously at work. In the intrauterine environment, the speed of maturation of the nervous and neuromuscular systems can be modified under the influence of the environment, whether internal or external, and genetic and extracellular environmental factors can interact. These processes continue from birth, depending on individual experience within a given environment, the constraints of that environment, and the educational attitudes of the people surrounding the child [4].

 From birth, the sensorial and motor systems are functional to differing degrees, coming into play in a certain order (cutaneous sensitivity, then vestibular, gustatory, olfactory, auditory, and visual); this occurs before the maturation of the nervous system is complete. Likewise, the motor behaviours of the newborn child are initiated from fertilisation. In the course of gestation, fully operational sequences of neuromotor and sensitive-sensory movements occur. These enable the newborn child and the infant to establish more refined sensory/motor “loops” or patterns which will be progressively integrated according to the myelinisation of motor pathways, motor abilities developing interdependently with affectivity and in interaction with the environment. Sensory-motor integration is thus constructed from the start of life *inutero*, in a gradual and exponential manner, at all levels of cerebral function, in the form of neurone networks, which in each individual constitute an enrichment and a reorganisation of the repertoire of human abilities [5, 8, 49] enabling since the birth, an intermodal sensorial transfer and between sensorial and motor modalities [50–53]. 

 The notion of cerebral functional plasticity is important from the very start of life; there is an individual functional plasticity, providing abilities for adaptation and regulation, that makes each individual unique. In this respect, “high-level potentialities” or exceptionally able children could be seen as having all sorts of potentialities available, and this could be directly related on the one hand, to the organisation of these neurone networks functioning particularly in intermodalities and on the other hand to conduction velocity rather fast.

 Psychomotor development data is still rare, since “gifted” children are generally noticed towards the end of their primary schooling by IQ measurement. In a recent French retrospective longitudinal studies [5], it has been obtained data concerning the psychomotor development of “high-potential” children (IQ ≥ 130 without significant difference between VIQ and PIQ) of a coming sample (*n* = 60) who at birth presented no health problem and no diagnosed pathology. It appears from this work that the particular maturity of their neuromotor and neurosensory systems and active tone enable early emergence of postural/locomotor acquisitions, hand-to-eye coordination, language acquisitions [9, 54], and cognitive processes, while it can be noted that this is in no way predictive of what these functions will become at school age. One of the oldest longitudinal studies [3] showed a higher percentage of boys, a greater frequency of first-borns, a higher family sociocultural status, greater average birth weight, and a larger number of breast-fed babies than among families with children of average ability; it also found advanced psychomotor development (including walking age one month earlier than the average, language acquisition three and a half months earlier than average, and early acquisition of reading).

 Certain other recent studies using retrospective surveys report advances in walking and language acquisition [55, 56], while the study by Louis [33] found no significant differences between a sample of gifted children and a control population. However, this study appears to present certain methodological data collection biases [33] because the data are collected on retrospective questionnaire.

 The unique French longitudinal data [5] evidence a specific pattern in developmental acquisitions among children with “high-level potentialities.” Among newborn infants, it was noted the ability for calm wakefulness of some duration (more than 8 minutes) compared to the average duration for newborn infants which is around 4 to 5 minutes according to Monod and Curzi-Dascalova [57]. There is rapid response to gaze and eye pursuit is good, through 90° to either side, already close to continual pursuit, involving both the eyes and the head. This exploratory mobility of the gaze gives these newborn infants an alertness accompanied by a certain sensitivity to all sensory perception in their environment (auditory, visual, olfactory, etc.). When the observer seeks to engage the gaze, there is a sort of livening of the infant's gaze, appearing to suggest particular alertness and attentional focus (involving the reticular formation), and these is more increase in adding the voice of the observer.

### 3.1. Neuro-Posturo-Locomotor Development

 The newborn postural responses obtained in this same study [5] show well-established neuromotor maturity in the synergy between extensor muscles (subcortex controlled) and flexor muscles (cortex controlled) strong axial tone enabling the child at birth to hold its head in the axis for at least two seconds and completely around one month. This marked maturation of the voluntary movement pathways undergoing myelinisation is attested by other neonatal indicators in the course of the first month of life, such as the relaxing of the upper limbs according to proximodistal criteria, active turning response (limb coordination), the disappearance of archaic reflexes (Moro reflex), walking reflex (around one month), and the Babinski sign (around 12 months). These findings attest an advance in cephalocaudal axial neuromotor maturation and in proximodistal maturation continues through the first years of life, enabling the child to rapidly acquire levels of coordination that will confer a degree of autonomy of movement. Generally speaking, with respect to posturo-locomotor acquisitions, it was noted an advance of at least one to two months, or one to two standard deviations above the average (see Table 1), these results being also found in a recent national survey of over 725 “high-level potentialities” children with 109 children prematurely born [58, 59]. There can however be a variability around the mean, so that it is possible that particular deviations may occur in development, related to cooccurring sensory or motor abnormalities [4].

 The interpretation of these advances in motor maturation among these children requires integration of several factors: the child's own motivation, his desire to make body movements, move about, acquire autonomy, or acquire power over his environment; there is also the child's environment in itself, which can encourage and provide incentive, or conversely put constraints on the child [24].

 This neuro-posturo-locomotor precocity should be distinguished from the sensory/motor advance noted in African children according to Vaivre-Douret [60] since this concerns only certain items that are fostered by the context and by cultural childcare practices and motor acquisitions that are specific to expected cultural norms [61–66].

### 3.2. Cognitive Development 

#### 3.2.1. Before 4 Years

With regard to cognitive development according to Vaivre-Douret [5], language precocity is of particular note (see Table 2), with babbling around four months on average and the imitation of animal noises around 22 months pointing to bucco-praxis skills. The first “sentence” appears around 18 months (the association of two words). These children later clearly enjoy clarifying words by series of synonyms, or opposites, or at a later stage by creating transitional neologisms, for instance by analogy or by playing on a concept. Mastery of language shows up in fluent use of words that are appropriate and therefore deliberate, implicating the acquisition of notions of spatial structuring (inside/outside, under/over, etc.) and of notions of temporal structuring (fast/slowly, yesterday/tomorrow, etc.) as can be seen in the appropriate usage of adverbs, verb tenses, and so forth. Later these children become curious of their environment, for instance, seeking to identify letters at an early age, recognising the written letters on posters and in newspapers, and so forth. They take an early interest in the meanings of words and in reproducing letters, so that around 34 months there are the beginnings of a form of spontaneous “writing” although the letters are not yet known.

 Lateral preference is established early [5], generally around 30 to 46 months, with a tonic functional predominance of the right half body. There appears to be a close link between left hemisphere predominance involved in the establishment of right-handed laterality and language precocity.

 Results of Vaivre-Douret's study relating to cognitive Piaget's tests such as the Casati and Lézine test [67] show that these children more rapidly reach maximum thresholds of sensory-motor intelligence (0–24 months), on average at least two months early. Likewise, these children perform particularly well in perceptive visuospatial activities (picture card matching, nested boxes, etc.).

 With regard to executive functions [5], there is also an advance of one or two years in age on average for planning abilities [68].

 It is striking that in the perceptive and cognitive fields all sensory perception (epidermic, tactile, gustatory, olfactory, auditory, and visual) appear particularly sharp, suggesting highly developed endogenous mechanisms and great receptiveness, enhancing sensory, emotional and affective reactivity, and a form of intuition functioning like a “sixth sense” thanks to intermodal abilities very sharp.

 With regard to cognitive functioning, excellent information-processing abilities can be noted (detection, perceptive discrimination, storage, and recall). Analytical processes are powerful (comparisons, matching features, and mental configuration). These abilities lead to ease and speed in understanding that facilitate the working memory, immediate memory being prominent in their daily functioning, for instance in what they observe (makes of cars, etc.). All of this endows them with an “elephant's memory” and an “eagle eye” according to Vaivre-Douret [4].

 These children, at a very early stage, look for strategies enabling them to understand situations, a sort of self-emulation functioning within their information processing system that is essential to their personal investment, which in return enables or facilitates the implementation of a targeted function. Thus, at an early age in the course of their psychomotor development, they may go through the transitional stages of coordination by discovering for themselves the strategies to achieve them, without those around them realising it, and very quickly reach the stages of sitting, standing, and walking.

 They can thus appear very early on as being “into everything,” eager, and curious, for instance, taking every object apart and exhausting their parents, all the more so because they are not interested in routine activities, they prefer complex games, and later on intellectual challenges, and they enjoy brain-teasers. They are always ready to experiment and innovate, they have creative skills, in particular in construction games, and, as early as two years of age, they take an interest in life and earth sciences, astronomy, metaphysics (life and death), and in books.

### 3.3. After 4 Years

If the stages of sensory-motor intelligence appear to occur early in the development of gifted children, achievements measured by Piaget-type formal operational tests (preservation of matter, classification, series, spatial representation) appear to follow a development process that runs closer to chronological age than to mental age, according to research conducted in the 1980s [69–77]. It thus appears that the rate of acquisition of the different developmental stages required for a certain skill can be faster in gifted children by way of their particular learning abilities and the implementation of a structural reorganisation, without the child in fact reaching the next stage any earlier. The neo-Piaget school [78–81] suggests that other mental processes are involved in controlling cognitive activity. This appears clearly in instances where there is the need to take account of perceptive criteria that are not familiar and that require elaboration of spatial relationships starting from the experience in hand. According to Lautrey [82], there may be interindividual variation in the cognitive processes used in the course of a Piaget-type task, and this variability could be related to the cultural, socioeconomic, and educational environment.

 This leads us to wonder about this possible discontinuity between sensory-motor stages reached quite early by these children and the need to reach the chronological age to realise operational potential. It is around 5 to 6 years that this heterogeneity appears to come into play, and there may be links with overinvestment of an intellectual nature, at the expense, in particular, of bodily stimuli or manual activities in the physical environment. This is reinforced by the fact that the child has reached school age, when the family may be more focused on intellectual performances (such as reading).

 In addition, on the basis of developmental data according to Vaivre-Douret [4, 5], at an early age, it can be noted that there is a fairly synchronous occurrence of functions arising from psychomotor and psychological development (motricity, language, socioaffective, and cognitive aspects). It is on this basis that we question the notion of “asynchrony” [12, 13] as a developmental trait among gifted children (i.e., the idea that intelligence, psychomotricity, affectivity, and sociability develop in a nonsynchronous manner). Indeed, if asynchrony does occur, it seems to emerge at a later stage, at school age, while previously psychomotor development was not observed to be delayed. This could then correspond to a deterioration of those functions that have not been used and not recognised (socially by the family and/or school and/or peers), or else it could result from the focalisation of functioning on a single, hyperinvested domain such as the cognitive domain, at the expense of the bodily and motor domains. Critical periods [83] or periods that are fertile in terms of incentive to learn can then to some degree be by-passed. Thus nonused functions run the risk of not developing sufficiently in the neuro-physio-psycho-social fields and lead on to malfunction, with possible repercussions on intellectual efficacy and the social and emotional behaviour of the child. According to Vaivre-Douret studies [4, 5, 58] on “high-potential” children, when performance IQ (subtests relating to body image, perception, spatial structuring, fine motor skills, lateralisation) show no significant differences in relation to verbal skill quotients, the children show better abilities for adapting to school and socially. Homogeneity between performance and verbal skill quotients appears to be an indicator of a “protective” factor.

## 4. Psychoaffective Development and Behaviour

 Among “high-level potentialities” children, if we refer to the main Freudian landmarks, the first stages of psychoaffective development come in rapid succession in “high-level potentialities” children, with a certain advance depending on the responses of the environment [4, 84]. There is an early awareness of the differences between the sexes and between generations around 30 months. If the child is left alone with his questionings, he is likely to experience a degree of confusion or distress in coping with these ideas, which can go as far as anxiety and lead to a depressive state or a denial of his own feelings. Because of the nature of their cognitive functioning, these children, as early as 3 years of age, possess a well-focused critical and self-critical sense, which may even be a hindrance or make them appear impertinent. They also have a sense of humour, which is characteristic of intellectual pleasure. In addition, they develop a form of generosity towards others, based on empathy and their desire to be accepted by sharing [4, 5].

 With regard to sleep, significant alterations did not note to the sleeping/waking cycle according Vaivre-Douret's study [5]. However when learning difficulties of the attentional or psychoaffective anxiety type occur, these children were observed to have difficulty getting to sleep or waking at night, and so forth. In a French national survey [58], at present being processed, a very significant statistical link between attentional disorders and sleep disorders have evidenced. Certain authors [85] have noted sleep disorders in a sample of early developers which, according to their data, can be linked to learning disabilities.

 It is important to emphasise that these “high-level potentialities” children have a highly active imagination which can rapidly become a source of anxiety if they are not reassured by their environment and if boundaries are not set.

 If the family and/or school environment, or some outside person, is not receptive to the child's needs and expectations—which may be completely out of step with “standard” demands—the child will show his distress or anxiety through behavioural or psychosomatic disorders, and by a lack of interest for learning, sometimes going as far as intellectual inhibition and reluctance to meet challenges. All of this leads to mental suffering of the psychoaffective type, generated by the inability to achieve fulfilment. If the environment only retains the notion of the child's advanced status in intellectual terms, drawing the child in this direction and only fuelling this aspect, and the child will become “bulimic” in proportion to his abilities to respond to this stimulation, triggering pleasure in this type of functioning which is itself reinforced by the fascination and the positive image of this pleasure experienced by the people in his environment. This happens via a form of sublimation and at the expense of his psychomotor and creative abilities, and this will lead him to isolate himself in an intellectual bubble, leaving the field open to his all-powerful imagination in a sort of “cognitive disharmony” as it has been termed by Gibello [86].

 The developmental data overall underlines the advantages of instating prophylactic guidance for “high-potential” subjects to accompany development in the psychomotor and psychological spheres. Early guidance could indeed avoid narcissistic withdrawal, behavioural deviance, and even personality problems (such as substance abuse disorders) or later decompensation episodes, in particular during adolescence, in the form of aggressiveness tending towards delinquency or depressive states accompanied by suicidal attitudes, and so forth [5, 49].

 A child carries with him an idealised self-based on parental images, but “high-level potentialities” children, who are highly empathetic, can form a hypertrophic “self-ideal” so as not to disappoint those around them and take refuge in intellectualisation. This sublimation becomes a defence mechanism against failure anxieties in case of parental inadequacy, whether conscious or unconscious. Thus, the child will renounce his emotional impulses (anger, worries, etc.) and his pleasures and fantasies by way of a sort of sacrifice. He will take refuge in failure or boredom, which can lead on to depression. Certain children settle into a sort of renouncement or inhibition towards play, which develops into a feeling of guilt that generates intractable anxiety. Thus, the child may organise his own neurosis as a form of defence, as seen in Rorschach's projective tests. Lebovici and Braunschweig [87], referring to the accumulation of sterile encyclopedic knowledge in some children, express surprise in finding obsessional mechanisms that were already well structured in these subjects, without there being any real anxiety. The study by Revol [88] evidences a marked trend to anxiety disorders in a population of early developers consulting in child psychiatry, observing phobias, and even obsessive-compulsive disorders.

## 5. Cognitive Functioning in “High-Level Potentialities” Children

 A certain amount of work in the fields of neurophysiology, neuropsychology, and anatomy affords a better understanding of the neurocognitive functioning of “high-level potentialities” children.

 The conduction velocity of nervous input has been studied by way of auditory stimuli, and faster transmission was shown for early developers compared with controls [89].

 According to some work, the relative and actual duration of REM sleep is greater among gifted children than among controls [90, 91]. In addition, a higher frequency of eye movement has been reported during REM sleep in this population [92, 93]. This data on REM sleep points overall to great cerebral plasticity and facilitation of memory processes. A large working memory capacity has also been shown across various studies [94–97].

 A link has been demonstrated between IQ and the “g” (general) intelligence factor [98–102]. However several studies on the speed of mental operations correlated with intelligence have suggested that performance in a reaction time task entails cognitive abilities, such as attentional abilities [64, 103, 104] and working memory [95–97]. The optimum use of these abilities enables better performance in reasoning tasks [105, 106]. Studies on cerebral activity using electroencephalograms suggest earlier physiological maturation [105, 106] with a lower alpha rhythm [107, 108].

 A certain number of studies in neuropsychology backed up by cerebral imagery enhance understanding of the specific characteristics of cognitive functioning in very bright children: response in habituation tasks, for instance, is faster [109, 110]. In addition, they have specific attentional abilities [64, 103, 104] and are able to screen out unsuitable information so as to avoid disturbance by perceptive distractors [111–113]. This leads certain researchers to conclude that “high-potential” children could be using a mode of information processing of the analogical type, enabling them to establish links between situations [71, 73, 75, 114, 115]. Thus these children are seen as having a mode of functioning that is different in qualitative terms from that of average children, with a faster processing speed on cognitive problem-solving tasks [116–118], as well as particularly marked learning abilities and an exceptional ability to transfer solving methods to a new situation [73, 75].This is thought to point to metacognitive abilities (knowledge of one's own mental functioning) in managing long-term memory (meta-memory) enabling a wider knowledge base than in other children by facilitating its encoding [115, 118–120].

 Certain authors [15] report a higher processing speed that they think is linked to the qualitative attributes coming into play in the processing of information (rich vocabulary range, attentional abilities, memory, cognitive mobility, and reasoning strategy, etc.). The prefrontal cortex (executive functions) is partly in charge of implementing these functions, which are mature in these subjects as can be seen from the considerable abilities of particularly gifted children in planning tasks, the Wisconsin classification test [121], and the completion of the Hanoi tower test [73, 115]. This implication of the prefrontal cortex is upheld by work using positron emission tomography (PET). Thus, in the study by Duncan et al. [122], it was observed that, in verbal and nonverbal tasks in an IQ test (saturated on nonverbal “g” factor), there was the activation of the lateral frontal area in both cerebral hemispheres. Likewise, another study [113] showed the activation of the left lower prefrontal cortex using PET when linguistic abilities and logical deduction were brought into play at the same time. Further to this, recent studies on hemisphere specialisation (dichotic listening, mental rotation, and functional MRI with hierarchical verbal stimuli) showed a degree of hemispheric equipotentiality in information processing for “high-level potentialities” children compared to controls [123–125]. However, other studies tend to show a greater implication of the right hemisphere among subjects particularly gifted in mathematics [126–128]. There is also thought to be a genetic factor determining the amount of grey matter in correlation with IQ, or even the proportion of white matter, according to Posthuma et al. [129]. Measurement of cerebral volume using anatomical MRI confirms this correlation between IQ and the volume of grey matter [130].

 Other studies in functional cerebral imagery using positron emission tomography (PET) show lower glucose consumption in the course of completion of verbal and nonverbal tasks [131, 132]. It can be hypothesised that lesser consumption of energy corresponds to a lesser activation of neurone networks, meaning less energy is required to complete cognitive tasks.

## 6. Learning Disabilities

 On the basis of our clinical experience over the last 20 years with “high-level potentialities” children, we have observed that the existence of out-of-the-ordinary cognitive abilities can also mask learning difficulties of neuropsychological (Table 3) or psychopathological origin (Table 4). 

 More there is significant difference between VIQ and PIQ, more there are learning disabilities. In a recent study, Loureiro et al. [133] have shown that highly gifted children with ADHD have a particular neuropsychological profile with an important difference (at least 20 points) between verbal IQ and performance IQ at Wechsler Intelligence Scale for Children (WISC III) when compared to highly gifted children without ADHD.

 Boys seem to be more frequently affected than girls [24, 58]. Male precocity is therefore more vulnerable but also carries a considerable advantage in the form of their cerebral plasticity, with abilities for processing information that enable them to use compensation or recovery strategies in an efficient manner.

 Indeed, it has not been demonstrated that prevalence of neuropsychological or neuropathological disorders among “high-potential” children is any higher than for average children [134, 135]. But the main difficulty resides in being able to assess the boundaries between what is “normal” and what is pathological, given the exceptional abilities for cognitive functioning (processing) among very bright subjects, where the strategies deployed can mask disorders that are nevertheless present.

 In attempts to describe students with learning disabilities who are gifted for example, [136, 137], there is a lack of consensus in the implications of giftedness and learning disabilities.

## 7. Conclusion

 Development data [5] evidences an advance in neurosensory-motor maturation among “high-level potentialities” children, both in postural, motor, and locomotor acquisitions, and in eye/motor coordination and attentional abilities. These results point to the reticular formation coming into play at an early stage in the form of awareness and attention and to rapid transmission speed of nerve input, as has been corroborated by different studies [89], leading to greater processing speeds [73, 116, 117]. The conduction velocity of nerve input could be related to specific conductivity characteristics, which could be explained at once by temporal characteristics of neurone decharging, linked to properties of the membrane and the synapse, and by the myelin surrounding the axons, combining to favour the speed of transmission of the electric signal.

 It can be hypothesised that these specific properties play an important part in encoding for memorisation (“elephant” memories among gifted children) and thus enable specific learning abilities with sensorial or/and motor intermodalities and, then, an extensive cerebral plasticity. These learning abilities are linked to processes of sensory integration, as underlined by the work by Planche [73] and Geary and Brown [138].

 The myelin sheath is a very good insulant for conduction, avoiding loss of the nerve input, and thus enabling the concentration of energy expenditure, which could explain the lower glucose consumption observed. This would mean that brain activity is more targeted, calling on only those regions required for processing the task, and thus decreasing energy demand in the brain metabolism, according to Neubauer [139]. This specificity relating to conductivity could account for high perceptive performances among these children (eagle eyes, fine hearing, etc.) by the fact that it activates more specific connections between neurones in both hemispheres of the brain, which themselves call upon specific neurone networks configured into populations or groups of neurones (functional architecture) according to neuroscience models [140]. 

 The “high-level potentialities” child could thus possess a specific form of cerebral functioning with a large information processing capacity at his disposal, which gives him great flexibility, considerable advantages in terms of learning abilities, and greater cerebral plasticity than the average child. 

 A recent research [59], in addition to this, shows an advance in neonatal anthropometric development (stature, weight, head circumference) for very gifted children born prematurely, whatever the gestational age. The results highlight a high proportion of hypertrophic premature infants in a sample of “high-level potentialities” children. Genetic and biologic factors could contribute in consequent manner to the features of this population of “high-level potentialities” children. Indeed, research is beginning to identify specific genes that govern the strong probability for a phenotype such as particular cognitive ability to be transferred by genetic factors [141–144].

 The contributions of all the work set out in this article point to the need to conduct fine analysis of the heterogeneous cognitive profiles of “high-level potentialities” children from a very early age and to realise the importance of preserving developmental continuity for the different functions, since these can prove very vulnerable, with the risk of becoming dissociated at a later stage on account of a specific plasticity, favourable or unfavourable environments (family, school, peers), or neuropsychological or neuropathological disorders that may develop [145]. It is important to take these factors into account to achieve fulfilment of the child's high potential, and to ensure that the child can invest his knowledge and skills in the best possible conditions, managing his impulses in a suitable manner, and identifying with a Self other than the idealised self-mediated by parental image.

 While a biological superiority appears to occur among gifted children, favourable conditions in the child's environment (sociocultural, educational, socioaffective, etc.) and good physical and mental health foster the realisation of the child's “high-level potentialities” or exceptional ability, and this is then accompanied by harmonious development of the personality. High-parental socioprofessional status is widely pinpointed in studies on high IQ populations [146, 147]. Moreover, the study of Vaivre-Douret [59] shows that, among children identified as gifted at school age, compared to those born full term, preterm infants showed a significant relationship between homogeneity in anthropometric variables and future motor and intellectual development when these children were exposed to a favourable perinatal environment (few pregnancy complications) and a favourable postnatal parental socioprofessional environment.

Indeed, intellectual superiority, as Ajuriaguerra [14], remarked does not necessarily lead to success in life: success needs to extend to the social, educational, professional, and affective spheres.

## Figures and Tables

**Table 1:** Comparison between observed motor development items in a sample of “high-potential” children followed longitudinally (*n* = 60) (Vaivre-Douret) [5] and French developmental standardised norms in the first two years of life [8, 9].

Items observed (Vaivre-Douret) [5]	Sample (*n* = 60) mean age and SD*(months, weeks)	DEF-MOT norms (Vaivre-Douret) [8] mean age and SD (months, weeks, days)	Brunet-Lézine norms revised [9] success 50%–90% (months)
Holds head in axis	1 m + 1 w	2 m 4 d + 1 m 1 d	3 m
Voluntary grasp	3 m + 1 w	4 m 10 d + 1 m 2 d	4 m
Turning overresponse	4 m + 4 w	6 m 10 d + 1 m 9 d	8 m
Sitting without support	6 m + 3 w	8 m 6 d + 1 m 2 d	10 m
Sits up alone	7 m + 3 w	8 m 24 d + 1 m 6 d	10 m
Stands up with support	8 m + 4 w	10 m 18 d + 1 m 18 d	10 m
Crawls	8 m + 3 w	10 m 12 d + 1 m 3 d	9 m
Takes bead between thumbs and forefinger	8 m + 2 w	9 m 10 d + 1 m 6 d	9 m
Independent walking	12 m + 4 w	14 m 20 d + 2 m 6 d	14 m
Start eating with a spoonon his own	12 m + 2 w	18 m 14 d + 1 m 2 d	17 m
		
Climbs stairs	15 m + 2 w	17 m 4 d + 1 m 10 d	—
Comes down stairs withhelp without alternating feet	16 m + 3 w	19 m 1 d + 1 m 2 d	—
		
Tower of at least 8 bricks	23 m + 4 w	29 m 1 d + 1 m 2 d	30 m
Climbs stairs alone without support alternating feet	24 m + 1 w	34 m 1 d + 2 m 1 d	—
		
Puts slippers on without help	24 m + 3 w	30 m 8 d + 1 m 5 d	30 m
Rides tricycle or bikewith stabilisers	24 m + 3 w	36 m 3 d + 1 m 1 d	—
		

*(Significant items (Student's *t*-test, liberty degree = 118), *P* < 0.001, compared to motor development standardised norms).

**Table 2:** Mean ages obtained for oral and written language development in a sample of “high-potential” French children in the course of the first three years of life compared to Brunet-Lézine norms.

Mean age obtained on items observed (Vaivre-Douret) [5]	Reference Brunet-Lézine EAP 1951 revised [9]	
Babbling (consonants): mean 4 months + 3 weeks.	7 months	8 months
First words (at least three): mean 9 months + 1 week.	12 months	10–17 months
Repetition of words in exponential manner from 12 months ± 4 weeks	18 months	17 months
First phrase (association of two words): mean 18 months ± 2 weeks	21 months	20 months
Imitation animal noises: mean 22 months ± 2 weeks	—	—
Accurate vocabulary, no baby talk language mature, easy, correct use of verb tenses: mean 22 months ± 2 weeks	—	—
Early spontaneous identifications letters and figures in the environment: mean 24 months ± 4 weeks	—	—
Enjoys giving synonyms or opposites: mean 28 months ± 4 weeks	—	—
Uses “I” (first person pronoun): mean 30 months ± 2 weeks	30 months	30 months
“Pretend” writing: mean 34 months ± 2 week	—	—

(—) No data from authors.

**Table 3:** Most frequent associated learning disorders among “high-potential” children.

Oral language disorders
Functional disorders:
Difficulty articulating
Stammer
Simple delay in using words
Simple delay in using language
Structural disorders
Dysphasia of the expressive type

Written language disorders
Dyslexia
Spelling difficulties
Dyscalculia

Developmental coordination disorders
Delay in posturomotor development and/or hand to eye
coordination
Developmental dyspraxia (VIQ > *PIQ*)
Ideomotor
Dressing
Visuospatial/visuoconstructional
Dysgraphia

Attention deficit disorder/hyperactivity and impulsiveness
(ADD, ADDH, ADHD)
Attentional dominant and/or hyperactive and/or impulsive
dominant

Specific psychomotor function disorders
Body image
Spatial organisation
Temporal organisation
Lateralisation
Neuromotricity (tone)
Tonico-emotional relationships

**Table 4:** Academic problems and behavioural and/or personality disorders among “high-potential” children.

Underperforming, poor student
Lazy, lacking motivation
Identified disorders including dysgraphia, dyslexia, spelling
problems, dyspraxia, attentional disorders, hyperactivity,
and impulsiveness
Intellectual/psychomotor/affective dyssynchrony
Clowning to gain attention
Destructuring tonus-emotion hyper-reactivity
Psycho-affective immaturity
Apathy
Frequent psychosomatic disorders
Behavioural fluctuation
Oversensitiveness
Withdrawing attitude
Anxiety
Willfulness and tantrums
Reactional aggressiveness
Violent behaviours
Delinquency, drug, and alcohol abuse
Megalomaniac trends
Difficulties of eye contact and difficult relationships
(borderline psychotic)
Depressive and suicidal tendencies
Identity disorders
Self-harm (mental or physical)
